# Nonlinear Modeling of E-Type Ferrite Inductors Using Finite Element Analysis in 2D

**DOI:** 10.3390/ma7085454

**Published:** 2014-07-25

**Authors:** Rosa Ana Salas, Jorge Pleite

**Affiliations:** Departamento de Tecnología Electrónica, Escuela Politécnica Superior, Universidad Carlos III de Madrid, Avda. de la Universidad, 30, 28911 Leganés (Madrid), Spain; E-Mail: pleite@ing.uc3m.es

**Keywords:** ferrite inductors, Finite Element Analysis (FEA), saturation effect, MnZn ferrites

## Abstract

We present here a modeling procedure for inductors with an E-shaped ferrite core valid for calculating the inductance of an equivalent circuit from the linear operating region to the saturation region. The procedure was developed using Finite Elements in 2D. We demonstrate that using a 2D section of the real core the results obtained are similar to the real ones, which solves the problem of convergence that appeared when E type cores were simulated in 3D, while also saving computational cost. We also discuss the effect of the gap-thickness on the magnetic properties. The data obtained by simulation are compared with experimental results.

## 1. Introduction

**Figure 1:**
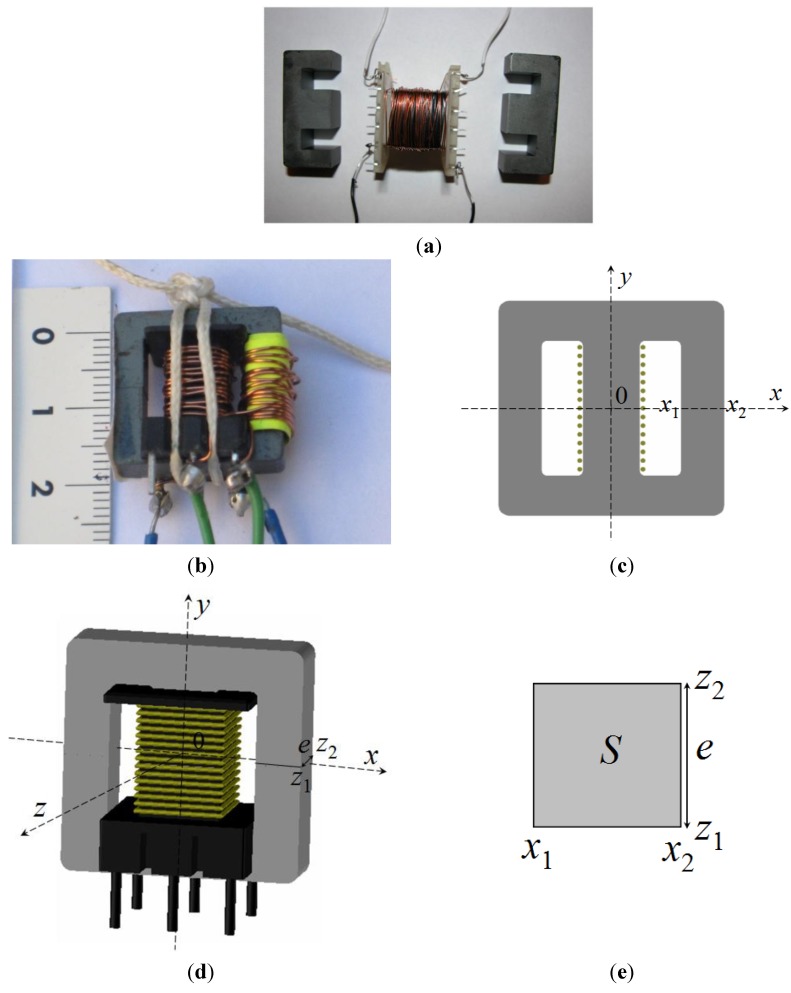
(**a**) Geometry of the real core; (**b**) constructed transformer with E20/10/5 core and two windings of 15 turns; (**c**) 2D computational domain; (**d**) inductor used in the 3D simulations; (**e**) cross-section *S* of the lateral column of the core in the *x*-*z* plane where *e* is the core thickness.

Modeling and simulation of ferrites in the field of power electronics requires obtaining parameters of their equivalent circuits [1,2,3,4,5]. Due to the nonlinear behavior of the core (linear, intermediate and saturation regions) [6,7,8], the actual losses, the different topologies (RM, POT, E cores) [9] and the number of turns, it is difficult to obtain a single, simple, and precise model valid for calculating the parameters of interest of the equivalent circuit such as inductance and resistance losses and which at the same time includes the effect of the gap-thickness on the magnetic properties. RM (Rectangular Modulus) cores arose due to the demand for coil formers with integrated pins that allow for efficient winding and high PCB packing densities. The shape of a POT core is round with an internal hollow that almost completely encloses the coil. Usually a POT core is made in two halves which fit together around a coil former. The literature oriented modeling and simulation of linear commercial ferrites is abundant [10,11,12,13,14,15,16,17]. However, to date there is a lack of studies that include all the working regions of the ferrite from the linear to the saturation regions. In this paper we focus on the inductors with E-type core. These inductors consist of two identical core halves, a coil former and a winding of copper wire (Figure 1a,b). The problem we found when simulating E type inductors in 3D is that there was no convergence for any size except for the smallest. This could not be solved, even by simplifying the geometry (right-angled corners) or eliminating the coil former in the simulation. There was no convergence either when including the air-gap. The solution we found was by performing 2D simulations.

In this paper we present a procedure for the E core that solves the convergence problem that occurs when solving the inductance calculations by 3D simulations. We have proven that by means of a 2D cross-section of the real inductor we are capable of obtaining results equivalent to the real ones reproducing the inductance curve from the linear to the saturation regions and even the geometrical effects of the air-gap. This has the advantage of greatly reducing the computational cost. It is also valid for all sizes of the core and the number of turns which makes it widely applicable. We investigate the accuracy of the procedure by comparing the experimental data with those computed by 2D Finite Element Analysis (FEA). In addition, we investigate the influence of the gap-thickness on the inductance curve.

This paper is organized as follows. In Section 2 we first describe the computational procedure that we used to carry out the simulations with the objective of obtaining the inductance curve from the linear to the saturation regions. We also describe the measuring procedure. After this we give a description of the E-type inductors that we used for the test. We also describe the measuring instruments that were needed to measure the nonlinear curves. Section 3 contains the study’s main results. We start by reporting results of the study of convergence. We next validate the 2D inductor model by experiment and by 3D simulations when convergence is achieved. Besides that, we analyze the effect of the air-gap on the flux and inductance curves. Finally, in the last section some conclusions are proposed.

## 2. Materials and Methods

### 2.1. Computational Method

Before presenting the procedure we begin by specifying the notation that is used in the rest of this paper. We use **B** and **H** (in bold) to denote the magnetic field vectors and |**B**| and |**H**| represent the moduli of the magnetic field vectors. We also use Ф to indicate the magnetic flux; *L* to represent the inductance; and *I* the DC excitation current value that flows through the inductor.

**Figure 2:**
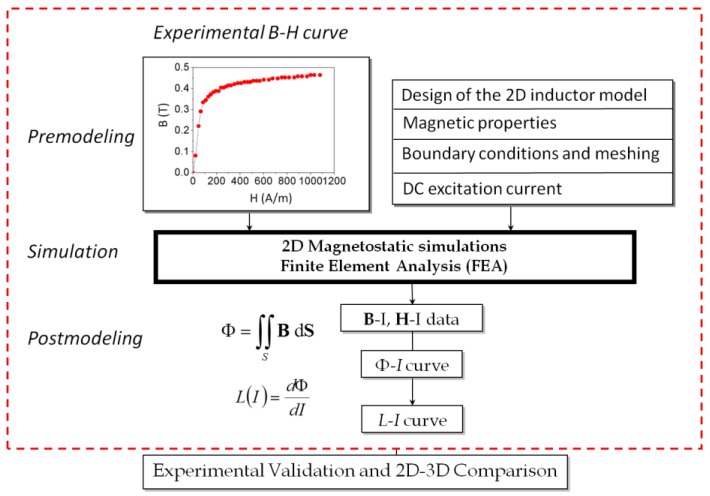
Simulation procedure.

In Figure 2 we show the procedure we used to obtain the *L*-*I* curve. The procedure combines experimental measurements with the use of Finite Element Analysis (FEA) in 2D. We also carry out simulations in 3D when convergence was achieved for comparison purposes. The FEA solution requires three steps: premodeling, simulation and postmodeling.

The first step of the simulation procedure is to take experimental measurements. These measurements are necessary to obtain the input parameters to carry out both the 3D and 2D FEA simulations (the *B*-*H* curve which characterizes the core material) and to check the validity of the obtained results (the *L*-*I* curve).

In order to obtain the *B*-*H* curve we built a two-winding toroidal transformer with the same material as the studied ferrite and we measured the Ф-*I* curve for current values from 0 to core saturation. We measured the magnetic flux with an electronic fluxmeter and provided the current with a DC power supply. We compute *B* and *H* values as:

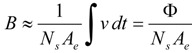
(1)

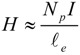
(2)
In these equations *N_p_* and *N_s_* are the number of turns of the primary and secondary winding respectively; *A_e_* is the effective area of the ferrite core; and *ℓ_e_* is the effective length of the ferrite. These effective dimensions are published in the manufacturers’ catalogs of commercial ferrites.

In order to obtain the *L*-*I* curve, we built a two-winding transformer with the same core as the studied one and we measured the Ф-*I* curve for current values from 0 up to core saturation. From this curve we derive the *L*-*I* curve by differentiation as *L* = *d*Ф/*dI*.

Next, in this same step of premodeling, we design the 2D and 3D computational domains (spatial regions that include the ferrite inductors). Then, we assign materials with the appropriate material attributes to each object in the geometric model of the inductor and define the boundary conditions. We assign to the coil former and winding their corresponding relative permeabilities µ_r_ and conductivities σ. We measure the *B*-*H* curve following the methodology previously described and assign it to the ferrite core. After we set material properties we assign the values of the DC excitation current from the linear to the saturation and the parameters to generate the mesh. For the 2D and 3D simulations we have chosen an adaptive meshing analysis. The adaptive refinement of the mesh consists of making it finer at the spatial points that are more irregular such as: corners, regions with irregular borders, *etc.* This technique reduces the computing time and the convergence and tolerance. This algorithm is implemented into the Maxwell program. We then specify the parameters related to the adaptative analysis to generate the mesh: percent refinement per pass, the number of requested passes to stop the algorithm and the percent error (τ).

For the *simulation process*, we used the Ansoft Maxwell field simulator’s magnetostatic solver (Ansoft Corporation, Pittsburgh, PA, USA). As outputs of the simulation process we obtain among others the distribution of the **B** and **H** fields of the inductor for each value of the DC excitation current *I*.

*Postmodeling* is the last step in a Finite Element Analysis. In this step we compute the magnetic flux evaluating the surface integral of the vector field **B** over a surface S of the core. Then, we obtain the inductance values by differentiation as *L* = *d*Ф/*dI*.

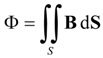
(3)


Finally, we validate the 2D results by comparing them with the experimental results and with 3D results when we achieve convergence.

### 2.2. Experimental Method

**Figure 3:**
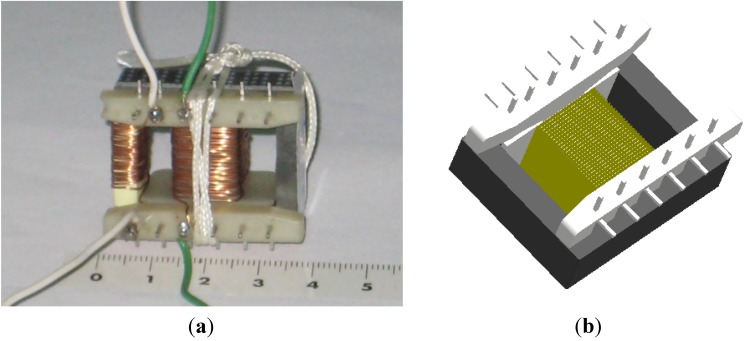
(**a**) Real transformer with E34/14/9 core and two windings of 28 turns; (**b**) E34/14/9 geometry used in the 3D simulations.

**Figure 4:**
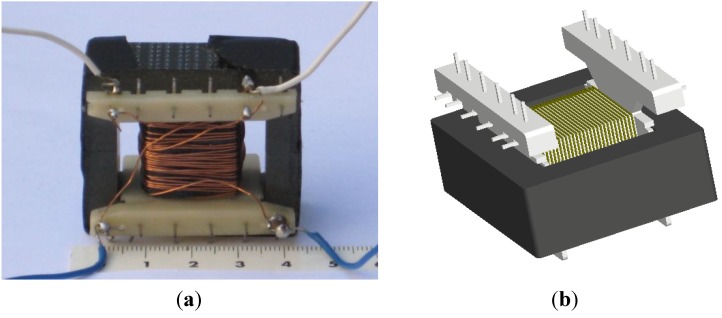
(**a**) Real transformer with E47/20/16 core and two windings of 28 turns; (**b**) E47/20/16 geometry used in the 3D simulations.

**Figure 5:**
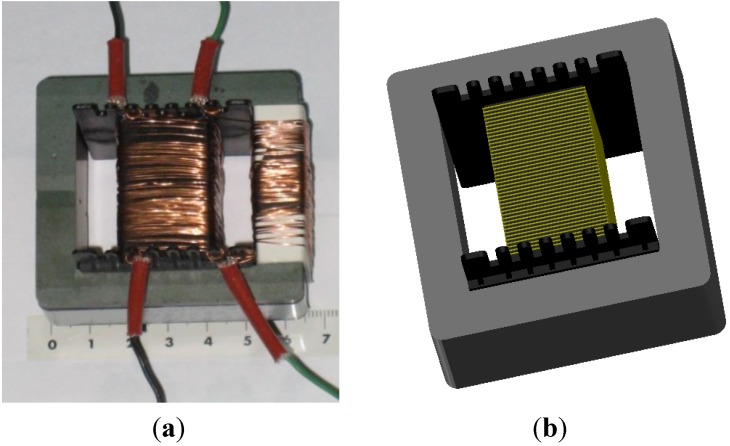
(**a**) Real transformer with E65/32/27 core and two windings of 45 turns; (**b**) E65/32/27 geometry used in the 3D simulations.

We studied MnZn soft ferrite cores made of 3F3 material from the manufacturer Ferroxcube. Their main application area is power transformers and inductors as well as general purpose transformers. This material has an initial permeability μ_i_ = 2000 ± 20% at 25 °C, a saturation flux density *B_sat_* ≈ 440 mT at 25 °C and *H* = 1200 A/m, Curie temperature *T_c_* ≥ 200 °C and a DC (direct current) resistivity ρ ≈ 2 Ωm at 25 °C. As commented before, the E-type inductor under study consisted of two identical core halves, a coil former and a winding of copper wire (Figure 1). We investigated four inductors with different sized E cores. These cores range, from smaller to larger: E20/10/5, E34/14/9, E47/20/16 and E65/32/27 cores (Figure 1b, Figure 3, Figure 4 and Figure 5). In order to carry out the experimental validation and according to Section 2.1 we first built four two-winding transformers. To do this, we first wound the copper coil around the central column of the core for the number of turns shown in Table 1. Then we wound a second winding around the lateral column of the inductor so that it works as a transformer (Figure 1b, Figure 3a and Figure 5a). As a result, the magnetic flux can be measured as a function of the excitation current. We built transformers with the same number of turns both in the primary and secondary coils. In order to measure the *B*-*H* curve we constructed a Ferroxcube TN23/14/7 with 60 turns in the primary and secondary coils (Ferroxcube Spain, Guadalajara, Spain). The number of turns we used was that needed to reach the saturation of the core. To measure the magnetic flux in the secondary coil we used the Magnet-Physik electronic fluxmeter EF4 from the MAGNET-PHYSIK Dr. Steingroever GmbH manufacturer (MAGNET-PHYSIK Dr. Steingroever GmbH, Köln, Germany). In order to generate the necessary DC current to excite the ferrite transformers we used a DC Xantrex power supply (Xantrex, Elkhart, IN, USA). This power supply has the following specifications: output voltage 0–7.5 V, output current 0–300 A and output power 2250 W. In order to measure the DC current *I* we used a Fluke 8010 A digital multimeter (Fluke Corporation, Everett, WA, USA). We also used a RFL voltage-current calibrator model 828 (Clarke-Hess Communication Research Corp., Medford, WI, USA). This calibrator supplies sinusoidal voltage to demagnetize the ferrite previous to the experiment.

**Table 1:** Four inductors with different sized E cores used for the test.

Size	Turns	Cylindrical Domain
E65/32/27	45	(*r*, *z*) ϵ [0, 40] × [−40, 40]
E47/20/16	28	(*r*, *z*) ϵ [0, 30] × [−25, 25]
E34/14/9	28	(*r*, *z*) ϵ [0, 25] × [−18, 18]
E20/10/5	15	(*r*, *z*) ϵ [0, 14] × [−15, 15]

## 3. Results

### 3.1. Convergence Analysis

We started the study by running 3D simulations with the four different sized inductors with different number of turns, shown in Table 1, from the manufacturer Ferroxcube. The E65/32/27 core is the biggest size and E20/10/5 is the smallest one. These inductors were modeled after the real ones, including windings, coil formers, and ferrite core with rounded edges (Figure 1d, Figure 3b, Figure 4b and Figure 5b). We ran the simulations with a standard personal computer (Hewlett-Packard Pavilion Media Center TV m7780n with a 2.13 GHz Intel Core 2 Duo Processor E6400 CPU, Hewlett-Packard Company, Palo Alto, CA, USA). We used the AutoCAD software to model the 3D inductor. In order to make the numerical simulations, we used the cylindrical domains (spatial regions which include the inductors) with the sizes shown in Table 1. The *x*, *y*, z coordinate system is centered in the geometric center of the core and the *z*-axis is perpendicular to the symmetry plane (Figure 1d). The first case under study is the case without air-gap (*g* = 0 µm). For the simulations we consider that the winding is made of copper (µ_r_ = 0.999991, σ = 5.8 × 10^7^ S/m), the coil former is made of hard rubber (µ_r_ = 1, σ = 10^−15^ S/m) and the ferrite core is characterized by the *B*-*H* curve of the 3F3 material. We measure this curve following the methodology previously described. To generate the initial mesh we assign τ = 1% and a DC excitation current *I* = 0.01 A. The simulations for the three first cases reported in Table 1 were not possible to complete as the program was not able to create the meshing. After running the program for approximately 30 h we noticed that it was not able to reach a solution, displaying the message “insufficient memory for module hnl3d”.

In contrast, for the E20/10/5 geometry (the smallest one), convergence was obtained and 87327 tetrahedra and a CPU time of 9 min and 7 s was necessary. To achieve convergence in the three first cases, simulations were repeated with simplified geometrical complexity using cores with right-angled edges and without the coil former. In this case complete convergence was not achieved either, solving only the conduction problem. In the case of the E20/10/5 core, where the program 3D converges, we studied the influence of the air-gap. To do this, we carried out a set of numerical simulations for two values of air-gap (*g* = 0 µm and *g* = 200 µm). For each value of *g* we used different DC excitation current values (*I* = 0.01, 0.02, 3, 8, 10 and 20 A). The values *I* = 0.01, 0.02 A are in the linear region and *I* = 3, 8, 10 and 20 A are in the saturation region. In Table 2 we show representative results where we can see that the introduction of the air-gap requires a greater number of elements and a greater computational cost.

**Table 2:** Computational cost for the 3D simulations as a function of the DC excitation current *I* (A).

Current	*g* = 0 µm		*g* = 200 µm	
*I* (A)	CPU Time	Tetrahedra	CPU Time	Tetrahedra
0.01	2 min 59 s	77280	11 min 58 s	250042
0.02	4 min 44 s	87327	11 min 59 s	250042
3	16 min 52 s	87327	24 min 46 s	250042
8	14 min 24 s	87327	33 min 28 s	250042
10	13 min 19 s	87327	52 min 22 s	250042
20	11 min 46 s	87327	1 h 8 min	250042

As a summary of the study of the convergence in 3D we obtained the following results. We made a study of convergence in 3D for four core sizes and the three larger ones did not converge either with or without coil former, or with simplified geometry. In the case of the small core E20/10/5, we studied the convergence and the computational cost for excitation currents in the linear and saturation regions showing that the introduction of an air-gap thickness requires, for this particular case, a greater number of finite elements and CPU time. Finally, in the small core E20/10/5 regardless of the air-gap thickness, the CPU time is less in the linear region than in the saturation region.

Once the calculation process 3D was finished, the 2D simulation was started with the aim of checking the convergence and influence of the gap-thickness on the magnetic properties. To do that we carried out the 2D simulations for the same four inductors using the new 2D inductor models with their corresponding square or rectangular domains, which are cross-sections in the *x*-*y* plane of the 3D inductors (see Table 3). As an example, in Figure 1c we can see the 2D inductor model corresponding to a 15-turn E20/10/5 inductor. Note that the 2D simulation considers that the inductor has an infinite depth. We carried out a set of simulations for *g* = 0 µm using different DC excitation values *I* = 0.01, 0.02, 3, 8 and 20 A. The values *I* = 0.01 and 0.02 A are in the linear region, and *I* = 3, 8 and 20 A are in the saturation region. We repeated these simulations for *g* = 2, 4, 6, 8, 10, 20, …, 100, 150 and 200 µm, achieving convergence in all cases.

**Table 3:** Computational domains used for the 2D simulations.

Size	Turns	Square or Rectangular Domain
E65/32/27	45	(*x*, *y*) ϵ [−40, 40] × [−40, 40]
E47/20/16	28	(*x*, *y*) ϵ [−30, 30] × [−25, 25]
E34/14/9	28	(*x*, *y*) ϵ [−25, 25] × [−18, 18]
E20/10/5	15	(*x*, *y*) ϵ [−14, 14] × [−15, 15]

**Figure 6:**
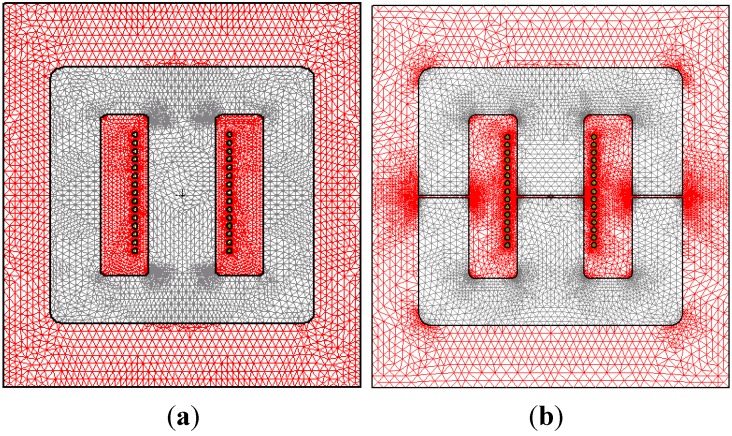
Mesh generated by the simulation program for the domain designed in 2D of the 15 turn-E20/10/5 inductor for τ = 1% and two different values of *g*: (**a**) *g* = 0 µm; (**b**) *g* = 200 µm.

For the E20/10/5 geometry (the smallest one), in Table 4 we show the CPU time and the number of elements generated in the domain by the program in 2D for three values of gap-thickness *g* (0, 50 and 200 µm) and for *I* = 20 A (saturation region). We take the percent error τ = 1% to carry out the simulations. As we only achieve convergence in 3D for this core, in the same Table we have also included the computational cost in 3D for comparison purposes. The most significant results are that convergence has been achieved both in 2D and 3D in all cases, with the computation time in 2D being in seconds and in 3D in hours. Another significant fact is that the gap-thickness increases as the computational cost increases in 2D and even more in 3D. In Figure 6a we show the mesh generated by the simulation program for the domain designed in 2D, where we can observe that the computation is carried out by adaptative meshing, so there is a higher number of triangles in some regions, specifically at the angles of the material. We also show the generated mesh for *g* = 200 µm (Figure 6b) in order to compare this with that obtained for the case *g* = 0 µm. We can observe in this case a greater concentration of triangles in the regions where the air-gap exists.

**Table 4:** Computational cost for the 2D and 3D simulations using the 15-turn E20/10/5 inductor.

Gap-thickness	CPU Time		Elements	
g (µm)	2D	3D	2D	3D
0	3 s	9 min	5894	68872
50	2 s	70 min	5705	241765
200	4 s	92 min	8822	250249

### 3.2. Experimental Validation and Influence of the Air-Gap

The validation of the design of the 2D inductor model and the analysis of the inductor behavior were carried out on the basis of:

(a) The distribution of the |**B**| and |**H**| fields on the surface and along the cross-section of the ferrite;
(b) The Ф-*I* and *L*-*I* curves, the influence of the gap-thickness on the Ф-*I* and *L*-*I* curves and experimental validation.

For (a) the 2D and 3D simulation data were compared to each other and in (b) the experimental results were included. For (a) the 2D and 3D simulations were carried out using the inductor with the smallest core E20/10/5 where convergence was achieved. We tested the validation with experimental measurements (b) for the four inductors of Table 1.

#### 3.2.1. The Distribution of the |**B**| and |**H**| Fields on the Surface and along the Cross-Section of the Ferrite

**Figure 7:**
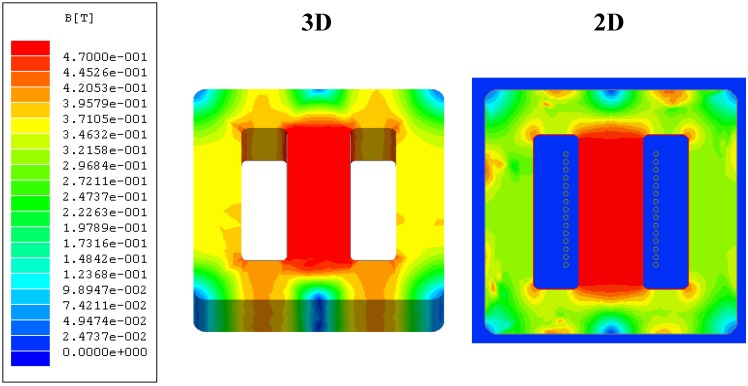
Values of |**B**| in 2D and 3D on the surface of the core. Results for *g* = 0 µm and *I* = 10 A (saturation).

**Figure 8:**
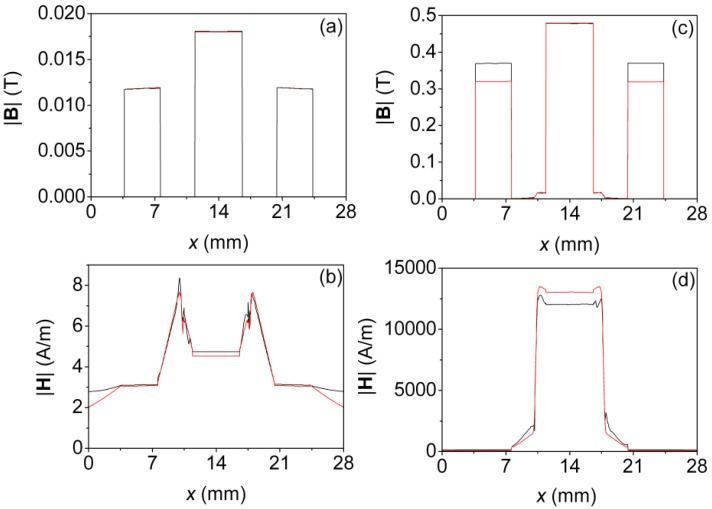
Results for *g* = 0 µm. Distributions of the |**B**| and |**H**| fields along the *x*-axis computed by 2D (—) and 3D (—) simulations, for (**a**,**b**) *I* = 0.01 A (linear region) (**c**,**d**) *I* = 10 A (saturation).

In Figure 7 we show the results obtained both in 2D and 3D of the distribution of the |**B**| field on the surface of the smallest ferrite core (E20/10/5, Figure 1c,d) in the case of saturation. In Figure 8 we plot the values of |**B**| and |**H**| along the *x*-axis computed by means of 2D and 3D simulations. In this Figure *x* = 14 mm corresponds to the center of the core. Figure 8a,b are the results for the linear region and Figure 8c,d for the saturation region. In the 3D simulations we chose the computational domain shown in Table 1 D: (*r*, *z*) ϵ [0, 14] × [−15, 15] (units are millimeters) as a cylindrical computational domain and τ = 1% as a fixed percent error. In the 2D simulations we chose the domain shown in Table 3 D: (*x*, *y*) ϵ [−14, 14] × [−15, 15] and τ = 1%.

The chosen 2D computational domain (cross-section of the real geometry) is capable of reproducing with precision the distribution of the |**B**| and |**H**| fields in the linear region and to a lesser extent in the saturation region. It can be observed in Figure 7 that both in 2D and 3D the magnetic field is not distributed uniformly on the surface of the inductor, varying for the simulation in 3D from 0.45 T in the central column (red) to 0.35 T on the lateral columns (yellow) and from 0.45 T in the central column (red) to 0.30 T on the lateral columns (green) in 2D. The smallest values (blue) are visible at the corners and edges that are farthest from the windings. As can be seen in Figure 8, as the excitation current is increased, the amplitude of the |**B**| and |**H**| fields increases, as is expected. The |**B**| and |**H**| fields are uniform in the section of the core in the linear, intermediate and saturation regions. As the ferrite is characterized by a high relative permeability (µ_r_ ≈ 2000) in comparison to vacuum (µ_r_ ≈ 1) most of the magnetic flux is practically confined in the core material. As a consequence, |**B**| and |**H**| are confined inside the core. The observed discontinuities in |**B**| correspond to the ferrite-air interfaces. As can be seen there is good agreement between the 2D and 3D results. We obtain almost identical values in 2D and 3D for |**B**| in the linear region at small current intensity (Figure 8a); nevertheless, in the case of saturation there are small differences in the values of |**B**| in the lateral columns as can be seen_in Figure 8c. In fact, the distribution of |**B**| in the lateral column is not the same in 2D and in 3D as can be seen in the slight color differences between the 2D and 3D images (Figure 7). These differences could be due to the way of computing **B** in the 2D case.

In the 3D case the spatial distribution of the *B_y_* field in the cross-section of the core (*x*-*z* plane, Figure 1c–e) is not totally uniform while in the 2D case it is supposed that *B_y_* is constant and uniform. Note that *B_y_* is the *y* component of the vector **B**.

#### 3.2.2. Influence of the Gap Thickness g on the Φ-*I* and *L*-*I* Curves and Experimental Validation

**Figure 9:**
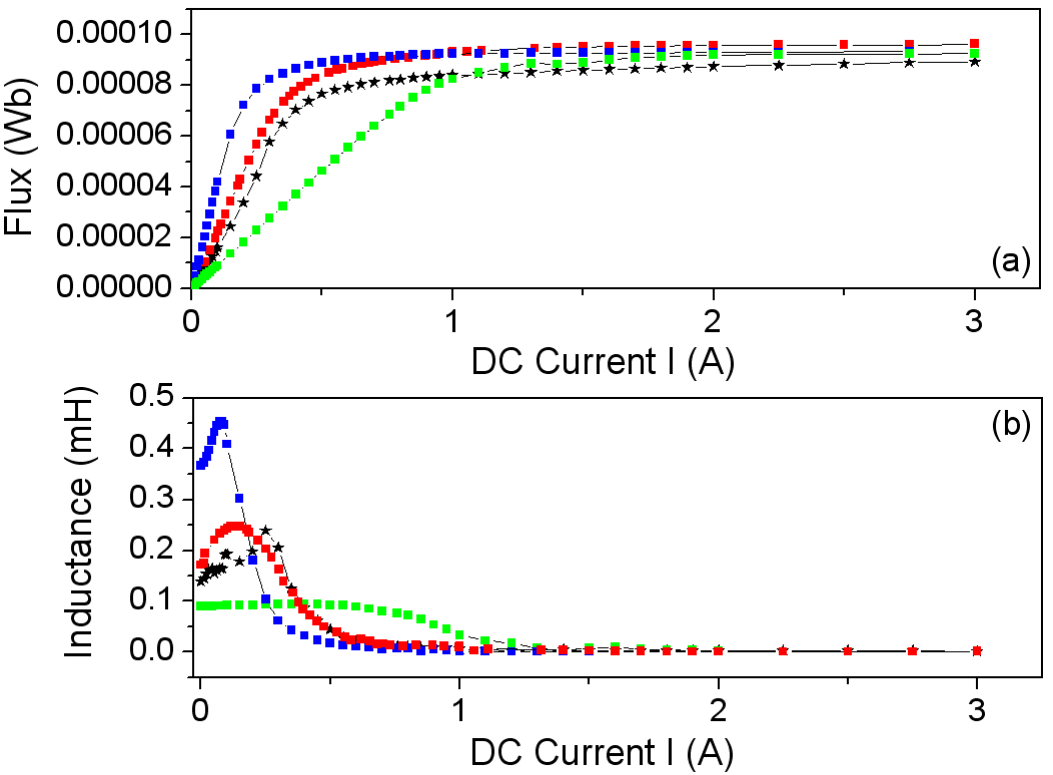
(**a**) Φ-*I* curves and (**b**) *L*-*I* curves computed by experiment (–∗–) and 2D simulations for *g* = 0 (–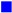
–), 5 (–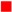
–), and 20 µm (–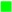
–).

The E inductor consists of two identical halves, their separation being adjustable according to the applications. Here only the case of direct contact (without divider) is analyzed. In order to find the air-gap thickness value that leads to the best agreement between the experimental and modeled curves and at the same time analyze the effect of *g* on the Φ-*I* and *L*-*I* curves, we carried out a set of 2D numerical simulations for various values of *g* using the smallest core (E20/10/5). In Figure 9 we show the results for *g* = 0 (without gap), 5 and 20 µm, showing that *g* = 5 µm has the best agreement with the experimental measurements. The equivalent value of this thickness can vary from one core to another, depending on the pressure of the clips, surface roughness and flatness, and so forth.

**Figure 10:**
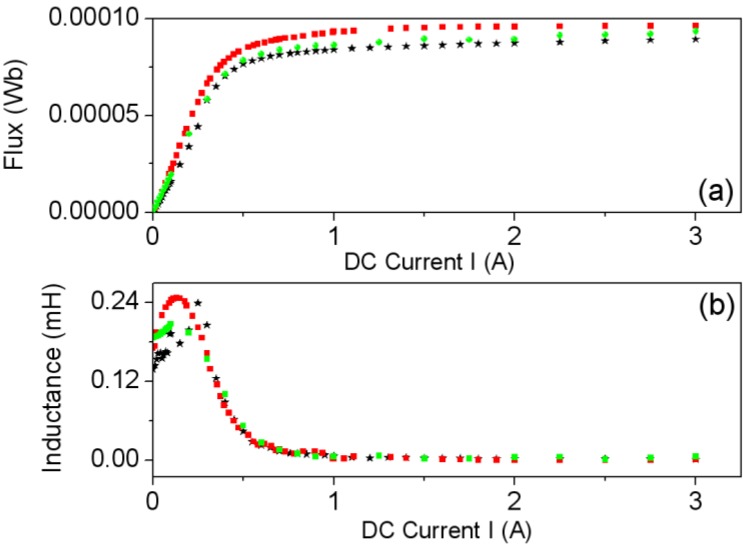
Results for the E20/10/5 ferrite core. (**a**) Φ-*I* curves and (**b**) *L*-*I* curves. Experiment (*), 2D simulations (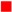) and 3D simulations (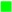) with *g* = 5 µm.

The Φ-*I* curves show three regions: *linear* (where the flux is proportional to the intensity of the current), *saturation* where it tends to an asymptotic value with a small slope and *intermediate*. By differentiating numerically Ф with respect to *I* we derive the *L*-*I* curve, obtaining a constant value of *L* in the linear zone, and a low constant value of *L* in the saturation region. In some inductors the Ф-*I* curve shows a slight change of curvature with an inflexion point in its linear region that leads to the *L*-*I* curve having a maximum in this region, instead of being constant. As the gap thickness increases, the slope of the Ф-*I* curve at low intensities (linear region) decreases, keeping the same asymptotic value of the magnetic flux in the saturation region. In the linear region, the gap thickness has considerable influence on the inductance value; as the thickness increases, the inductance values decrease considerably and the range of the excitation intensity increases. To check the validity of the 2D model we compared the results to 3D simulations and with experimental measurements, showing again a good agreement between all the results for *g* = 5 µm (Figure 10). Both the 3D and 2D simulations show a very good agreement with the experimental curve. The small differences could be due to the way of computing Φ, evaluating the line integral of the *B_y_* field of the Equation (4)—instead of evaluating the surface integral of Equation (3)—and supposing that *B_y_* is constant in the direction of the core thickness. In order to compute Φ we chose the surface *S* = [*x*_1_, *x*_2_] × [*z*_1_, *z*_2_] in a lateral column in the core (see Figure 1c,d). These cores have a rectangular section lateral column. In Equation (4) *e* is the core thickness as shown in Figure 1c,d.

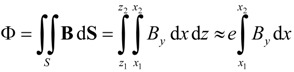
(4)


**Figure 11:**
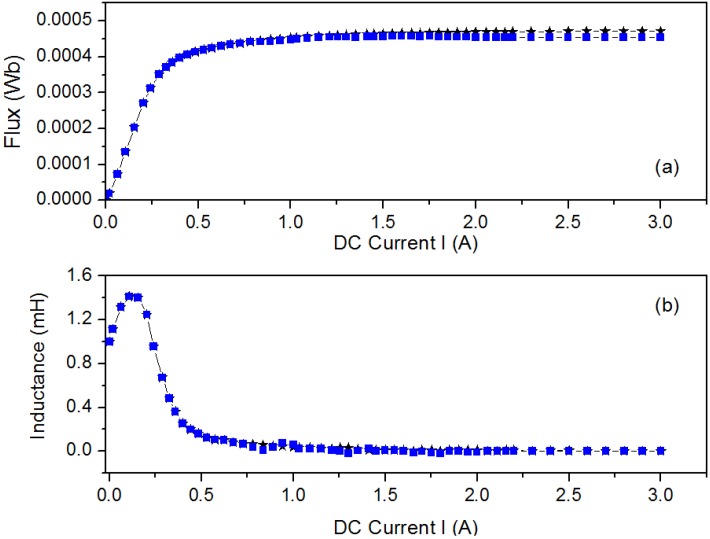
Results for the E34/14/9 ferrite core. (**a**) Φ-*I* curves; and (**b**) *L*-*I* curves. Experiment (–*–) and 2D simulations (–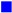
–) with *g* = 5 µm.

**Figure 12:**
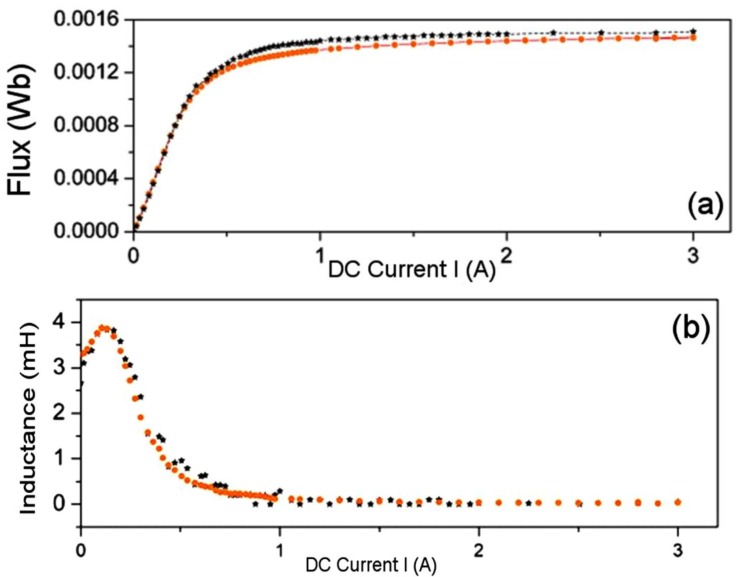
Results for the E47/20/16 ferrite core. (**a**) Φ-*I* curves and (**b**) *L*-*I* curves. Experiment (–*–) and 2D simulations (–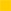
–) with *g* = 4 µm.

**Figure 13:**
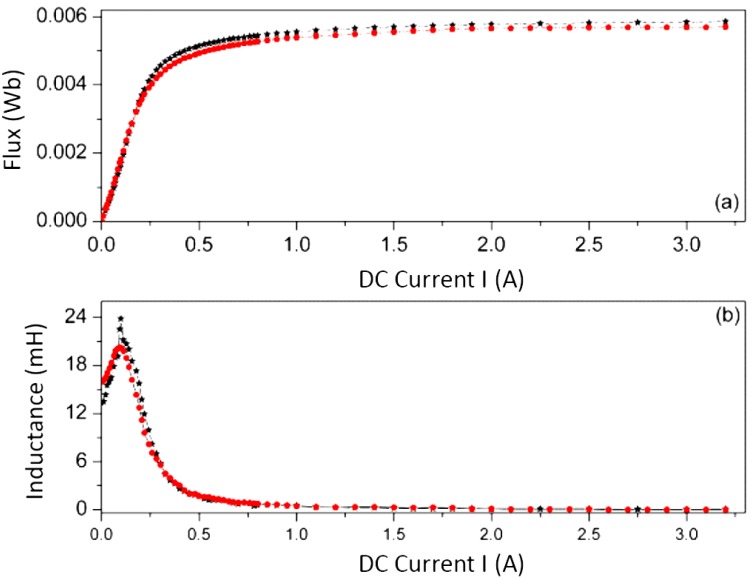
Results for the E65/32/27 ferrite core. (**a**) Φ-*I* curves and (**b**) *L*-*I* curves. Experiment (–*–) and 2D simulations (–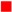
–) with *g* = 0 µm.

In order to complete the study, we tested the core sizes shown in Table 3, and as an example we show the Φ-*I* and *L*-*I* curves for the E34/14/9 (Figure 11), E47/20/16 (Figure 12) and E65/32/27 (Figure 13) cores obtained by 2D simulations and experiment. The 3D simulations are not included as there was no convergence. Again, there is good agreement between the experimental and 2D results, leading to the validation of the 2D simulations. As we were able to check, the same effect of the gap-thickness on the curves was obtained. The gap-thickness values obtained by 2D simulation that lead to the best agreement with laboratory experimental results are *g* = 0 µm for the largest core E65/32/27 and from *g* = 4 to 5 µm in the cores E47/20/16 and E34/14/9, respectively. As happened with the E20/10/5 core, the *L*-*I* curve shows a maximum at low current values. The maximum of the inductances are 20 mH for the E65/32/27 core; 4 mH for the E47/20/16 core and 1.5 mH for the E34/14/9, which decrease as the size of the core decreases.

## 4. Conclusions

In this paper we proposed a specific procedure to calculate the inductance in a ferrite inductor with an E-type core. The modeling procedure includes a new 2D computational domain for inductors with an E-type ferrite core, equivalent to the real core, which was validated comparing the Φ-*I* and *L*-*I* curves of different sized cores. It was also validated by comparing the distribution of the moduli of the **B** and **H** fields on the surface and along the cross-section of the ferrite, both in 2D and 3D. The 2D domain is based on a cross-section of the real inductor and was tested for the case of small gap-thickness comparing the Φ-*I* and *L*-*I* curves to the corresponding curves that were simulated in 2D, since in 3D, convergence could not be reached except for the smallest inductor. The 2D model is capable of reproducing with precision the behavior in all working regions except in the saturation region where we find slight differences. In addition, we confirmed that the procedure is capable of reproducing the effect of the gap-thickness on the magnetic properties. Another advantage of the procedure is that with one measurement of the *B*-*H* curve, all other sizes, number of turns, *etc.* can be modeled.
